# Effectiveness of Video-Game-Based Therapy to Improve Hand Function in Children with Cerebral Palsy: A Systematic Review and Meta-Analysis

**DOI:** 10.3390/jcm13247524

**Published:** 2024-12-11

**Authors:** Mátyás Vezér, Orsolya Gresits, Marie Anne Engh, Bence Szabó, Zsolt Molnár, Péter Hegyi, Tamás Terebessy

**Affiliations:** 1 Centre for Translational Medicine, Semmelweis University, Üllői út 26., 1085 Budapest, Hungary; vezer.matyas@semmelweis.hu (M.V.); gresits.orsolya@semmelweis.hu (O.G.); engh.marie@semmelweis.hu (M.A.E.); szabo.bence1@semmelweis.hu (B.S.); molnar.zsolt1@semmelweis.hu (Z.M.); hegyi.peter@semmelweis.hu (P.H.); 2 Department of Orthopaedics, Semmelweis University, Üllői út 26., 1085 Budapest, Hungary; 3 Department of Anaesthesiology and Intensive Therapy, Semmelweis University, Üllői út 26., 1085 Budapest, Hungary; 4 Department of Anaesthesiology and Intensive Therapy, Poznan University of Medical Sciences, 61701 Poznan, Poland; 5 Institute of Pancreatic Diseases, Semmelweis University, Üllői út 26., 1085 Budapest, Hungary; 6 Institute of Translational Medicine, University of Pecs Medical School, Szigeti út 12., 7624 Pecs, Hungary

**Keywords:** video-game-based therapy, cerebral palsy, grip strength, grasp function, manual dexterity, hand function questionnaire

## Abstract

**Background:** Advances in technology have led to the emergence of new therapeutic methods such as video-game-based therapy (VGBT). This may be a promising new method for improving upper limb function, but the role and proposed uses still need to be clarified. This study aims to investigate the effectiveness of VGBT in children with cerebral palsy (CP) compared to conventional therapy (CT). **Methods:** A systematic search of five databases was conducted (Cochrane, Embase, Pubmed, Scopus, Web of Science) in January 2024 to identify randomized controlled trials that compared VGBT interventions with CT for children with CP. Our primary outcomes focused on tests on hand functions (HFs) (grip strength, grasp function, manual dexterity tests, hand function questionnaires tests, and upper limb fine motor function tests). A random effects meta-analysis was performed, and ROB2 and GRADE tools were used. **Results:** Of 2882 articles reviewed, 22 were selected, involving a total of 785 children. Three outcomes were statistically significant in favor of the intervention group: for grasp function, the standardized mean difference (SMD) was 0.80 (95% confidence intervals 0.06, 1.55); for HF questionnaires, the SMD was 0.36 (95% CI 0.04, 0.68); and for HF tests, the SMD was 0.42 (95% CI 0.03, 0.81). The ROB was high risk in two, some concerns for four and low risk in the other cases. The GRADE was moderate in two, while the remaining were low and very low in half of the papers. **Conclusions:** VGBT has the potential to be an effective tool for rehabilitation of disabled upper limb function in CP as an adjunct to other traditional forms of therapy by integrating it into everyday rehabilitation.

## 1. Introduction

Nearly 50% of patients with CP suffer from impaired arm and hand function, leading to limitations in activities and participation [1,2]. The primary objective of rehabilitating upper extremities in children with CP is to mitigate the loss of function caused by brain injury. Specifically, this involves leveraging the neuroplasticity of the brain to improve hand function [3]. For children with movement disorders such as CP, rehabilitation takes up a significant part of their lives. Playful tasks can enhance cooperation in children during rehabilitation therapies, and games and playful elements are often included in upper limb rehabilitation exercises for children with CP, such as Occupational Therapy [1,4] and Constraint-Induced Movement Therapy (CIMT) programs [5], as they can lead to better outcomes. Advances in technology have led to the emergence of new therapeutic methods such as video-game-based therapy (VGBT), which may be a promising new method for improving upper limb function. Potential benefits of video-game-based therapy include the relatively low cost of commercial gaming consoles, the possibility of repeating functional tasks several times, the flexible nature of the virtual environment with varying levels of difficulties, the provision of sensory and cognitive input via visual, auditory, and occasionally tactile signals, along with feedback and the ability to enhance patient motivation [3].

Although VGBT theoretically contains all the essential components needed to promote or stimulate neuroplasticity, it remains to be seen whether this translates to successful practical application [6]. Numerous studies report the benefits of video-game-based therapy in the rehabilitation of individuals with neurological impairments; however, few studies examine the effectiveness of video-game-based therapy in children with cerebral palsy (CP) [7,8]. In a crossover study, a greater improvement in upper limb motor skills was observed; however, this did not mean a significant improvement in everyday activities compared to the control group [3].

These potential benefits associated with video-game-based therapy may lend themselves to increasing the effectiveness and compliance of therapeutic forms alone or in combination. Nonetheless, the specific role and recommended usage of VGBT still need to be determined.

The purpose of this study was to estimate the potential effect of VGBT in the rehabilitation of the upper extremity in children with cerebral palsy by reviewing and synthesizing the existing data in the literature.

## 2. Materials and Methods

The authors conducted this systematic review and meta-analysis following the guidelines outlined in the Cochrane Handbook [9] and followed the PRISMA 2020 guidelines [10]. The study protocol was registered on PROSPERO (CRD42022312513).

### 2.1. Search Strategy

A systematic search was performed in five databases: MEDLINE (via PubMed), Cochrane CENTRAL, Embase, Scopus, and Web of Science. The original search was conducted on 27 April 2022 and was updated on 26 January 2024, with the following search key:

Cerebral palsy AND (virtual reality OR game OR nintendo OR playstation OR xbox).

The authors (OG and MV) searched all fields in all databases except in Scopus, where only titles, abstracts, and keywords were searched. References of eligible studies were later also searched for further potentially eligible studies.

### 2.2. Study Selection

After removing duplicate articles, two of the authors (OG and MV) independently conducted the selection process: first, titles and abstracts were screened.

If an article met the inclusion criteria or if there was any uncertainty about its eligibility, the authors (OG and MV) obtained the full text of the article. The remaining full texts were also reviewed in a similar manner. The degree of agreement was measured by calculating Cohen’s kappa values. Any disagreements were resolved by a third author (TT).

### 2.3. Inclusion/Exclusion Criteria

Only randomized controlled trials published in peer-reviewed journals were considered, and all other study types were excluded. The population had to consist of children with CP under 18 years of age, and at least one study group had to receive VGBT with or without accompanying traditional physical therapies, partially eligible studies were excluded.

### 2.4. Data Extraction

A standardized data collection sheet was created in Microsoft Excel. Data collection was performed by one author (MV) and reviewed by another (OG). The following outcomes were extracted: grip strength, grasp function, upper limb fine motor function, WeeFim self-care, hand function questionnaire, manual dexterity, the Jebsen Taylor hand function, and ABILHAND-Kids. Any discrepancies were resolved by discussion. We extracted data from upper extremity function tests for manual dexterity separately and then expanded this by pooling them together with physical tests which aim to examine upper extremity function, in order to gain a more accurate understanding of upper limb functions. In cases where multiple tests were presented in the same study, we chose the one that contained the most cases. We also examined the questionnaires on upper extremity functions separately. Data types extracted from each included study are presented in Table S4.

### 2.5. Assessment of Evidence Quality and Risk of Bias

Two independent reviewers (OG and MV) evaluated the risk of bias using the Cochrane risk-of-bias tool for randomized trials (ROB2), and the quality of evidence with the Grading of Recommendations, Assessment, Development, and Evaluations (GRADE) scale [11,12]. Disagreements between the two authors (OG and MV) were resolved by discussion.

### 2.6. Statistical Analysis

The authors (OG and MV) conducted a meta-analysis for each outcome when at least three studies were available and presented the results using Forest plots. To assess differences between the groups being compared, standardized mean differences (SMDs) and their corresponding 95% confidence intervals [13] were calculated for continuous outcomes [14].

The change in mean was calculated by subtracting the mean before treatment from the mean after treatment. The standard deviation of change (as correlation was not available) was calculated using a conservative approximation, i.e., we assumed a correlation of −1. Different research studies use different rating tools to assess identical outcomes; therefore, it is not feasible to combine the mean differences from different randomized controlled trials (RCTs). In order to obtain a common outcome, we divided the results by their corresponding standard deviations (SDs) to derive an effect size referred to as the standardized mean difference (SMD). The SMD results obtained in this way can be pooled in the meta-analysis because the unit is the same in all studies. The standardized mean difference (SMD) of 0.2 is considered small, 0.5 medium, and 0.8 large [14].

The Q-profile method was used to estimate heterogeneity variance by calculating the measure τ^2^. The Cochrane Q test and I^2^ values were used to evaluate statistical heterogeneity across trials where a *p*-value of less than 0.1 was considered statistically significant. Due to the low number of available studies, Egger’s test for small-study effects could not be performed. Publication bias was assessed using funnel plots.

Statistical analyses were conducted using the R programming language, specifically with the “meta” package (v5.2.0) developed by Schwarzer in 2022 and the “dmetar” package (v0.0.9000) developed by Cuijpers, Furukawa, and Ebert in 2020 [15,16].

## 3. Results

### 3.1. Study Selection

**Figure 1:**
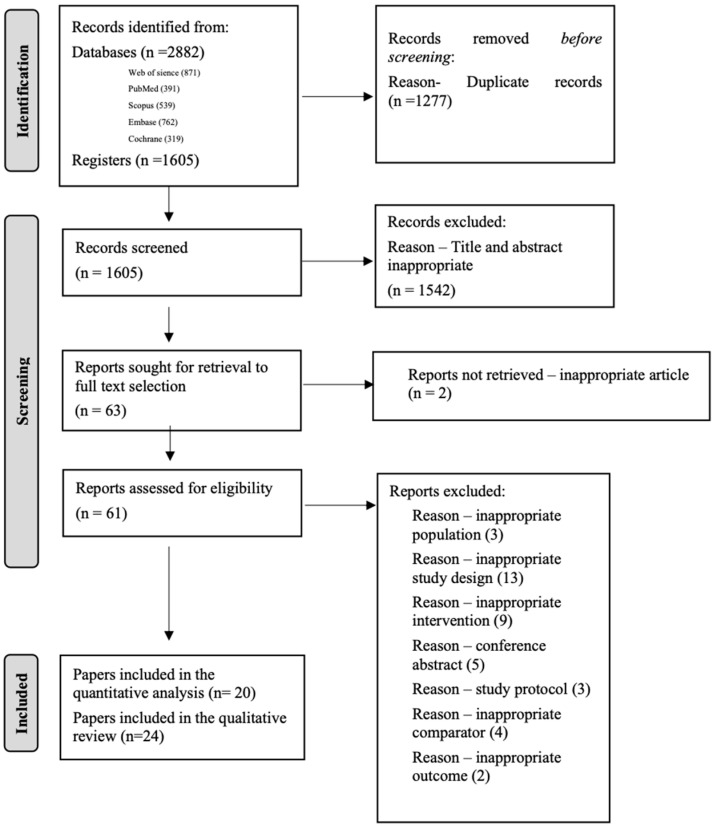
PRISMA 2020 flow diagram detailing the selection process.

A total of 1605 articles were screened, 63 full-texts were obtained, and 24 papers were finally identified as eligible. Cohen’s kappa was 0.96 for title and abstract selection, and 1 for full-text selection. The selection process is detailed in the PRISMA flow diagram in Figure 1.

In one of the articles [17], the age range was from 5 to 20. We reached out to the authors of the article to inquire how many children were above the age of 18, but did not receive a response. Considering that the control group consisted of 10 participants with an average age of 12.4 ± 4.93 years, and the intervention group also had 10 participants with an average age of 10.6 ± 3.78 years, assuming a normal distribution, only one individual in the control group would have exceeded 18 years based on our calculations. We chose to consider this to be within a reasonable margin of error and included the study regardless.

Out of 24 articles [6,7,8,12,18,19,20,21,22,23,24,25,26,27,28,29,30,31,32,33,34,35,36,37], 2 could not be included in the meta-analysis: as they reported none of our outcomes of interest [18,19]. Two further articles were suitable only for the review section [3,20]: one article could only be included in the systematic review section due to limited reporting of outcomes in the setting of a cross over study design [3], and another article was incorporated only into the review section because the only outcome of interest (self-care score) was not sufficiently comparable to self-care scores used in other included studies [20].

### 3.2. Basic Characteristics of Studies Included

The baseline characteristics of the studies included are detailed in Supplementary Table S1. A total of 785 children aged 3 years to 20 years were involved in the investigations. Patients were hemiparetic in ten articles [20,21,22,23,24,25,26,27,28,29] diplegic in two [30,31], mixed in five studies [17,32,33,34,35], and unknown in four [36,37,38,39]. The studies included lasted from 3 to 16 weeks. In ten papers, the intervention group received Nintendo Wii alone or with other therapy, and VR intervention was applied exclusively or with other therapy in eight cases. In the remaining articles, leap motion controlled (LMC) games or computer-game-based systems were used. The control groups included neurodevelopmental training (NDT), conventional treatment (CT), resistance training (RT), CIMT and Hand Arm Bimanual Intensive Therapy (HABIT), OT, exercise therapy (ET) or no treatment. In all articles, the intensity of therapies applied was similar and therapy duration was similar, except for five studies in which it was increased in the VGBT groups [21,22,24,26,33]. Detailed information on intervention and comparator is also summarized in Supplementary Table S1.

The risk of bias results is detailed in Supplementary Table S2. The overall risk of bias was rated as ‘high risk’ for 2 articles [25,35] and ‘some concerns’ for 4 articles [21,22,30,31], and the remaining 14 were judged as ‘low risk’ [17,23,24,26,27,28,29,32,33,34,36,37,38,39].

The GRADE assessment showed ‘very low’ evidence for three (self-care, grip strength, and upper limb fine motor function) out of eight, only ‘low’ in three other (grasp function, manual dexterity, and Abilhand-Kids) outcomes and ‘moderate’ in two cases (hand function questionnaire, and Jebsen Taylor hand function) (see Supplementary Table S3).

### 3.3. Systematic Review

**Grip strength:** Six of the chosen articles reported grip strength [23,24,25,32,34,39] (Table 1). The improvement in grip strength was found to be significant in two articles [24,34], and another two found it to be insignificant in the VGBT group compared to the improvement in the control group [23,25]. Only one paper reported an insignificant change in favor of the control group [39], and one found no difference between the two groups [32]. On the basis of the risk of bias assessment, five studies were ‘low risk’ [23,24,32,34,39] and the remaining one was ‘high risk’ [25] (see Supplementary Table S2).

**Grasp function:** The effect of video-game-based therapy on grasp function was investigated in nine studies [3,17,21,24,26,31,34,37,38] (Table 1). In the VGBT group, seven out of eight articles found improvements compared to the control group [17,21,24,26,31,34,38], but only four of these were significant [24,31,34,38]. Risk of bias was rated ‘some concerns’ for two studies [21,31] and ‘low risk’ for six studies [17,24,26,34,37,38] (see Supplementary Table S2). The one remaining crossover study that we were unable to use in the meta-analysis showed a significant improvement in favor of the VGBT group [3].

**Manual dexterity physical test:** Changes in manual dexterity were evaluated in ten papers [17,21,22,23,25,26,28,29,33,39] (Table 1). Six articles found no between-group differences [17,23,28,29,33,39], three described minor differences, two of which favored VGBT [25,26], and one favored the control group [21]. Statistically significant changes were reported only in one case favoring VGBT [22]. The risk of bias was rated ‘low risk’ in seven cases [17,23,26,28,29,33,39], one was classified as ‘high risk’ [25], and another one as ‘some concerns’ [21,22] (see Supplementary Table S2).

**Upper limb fine motor function:** Improvement in upper limb fine motor function after video-game-based therapy was assessed in 17 articles [3,17,21,22,23,24,25,26,28,30,31,33,34,36,37,38,39] (Table 1). Seven studies found no differences between the groups [17,23,28,33,36,37,39], eight articles found a greater improvement after VGBT [22,24,25,26,30,31,34,38], five of which were significant [24,30,31,34,38], and one paper in favor of the control group, albeit mathematically insignificant [21]. The results of the risk of bias assessment were classified as ‘low risk’ in 11 papers [17,23,24,26,28,33,34,36,37,38,39], ‘high risk’ in 1 study [25] and ‘some concerns’ in the remaining four [21,22,30,31] (see Supplementary Table S2). Only one study, which we were unable to include in the meta-analysis, found a significant improvement in the VGBT group in Quality of upper extremity skills test (QUEST) score [3].

**Hand function questionnaires:** Seven articles assessed hand function with questionnaires [21,22,25,26,28,29,39] after video-game-based therapy (Table 1). Three of them showed an insignificantly better [25,26,39] and one a significantly [22] better improvement after VGBT, and the remaining three cases showed no differences between the investigated groups [21,28,29]. In four out of seven studies, the risk of bias was judged as ‘low risk’ [26,28,29,39]; of the remaining three, one was classified as ‘high risk’ [25] and two as ‘some concerns’ [21,22] (see Supplementary Table S2).

**Self-care (WeeFim):** The WeeFim self-care subdomain was reported in three studies [21,27,35] (Table 1). All articles showed a better improvement in the VGBT group; however, this difference was significant only in one study [21]. The risk of bias was judged as high risk for one study [35], low risk for another study [27], and some concerns for the third study [21] (see Supplementary Table S2). Of the articles not suitable for meta-analysis, one study [20] found an insignificant improvement in Pediatric Evaluation of Disability Inventory self-care domain in favor of the Nintendo Wii group.

**Abilhand-Kids:** ABILHAND-kids was used in seven papers to measure hand function with questionnaires [3,21,22,25,26,28,29] (Table 1). Three papers found no differences between the groups [21,28,29], two others found an insignificant [25,26] and one a significant [22] improvement in the VGBT group compared to controls. Risk of bias in half of the studies was ‘low risk’ [26,28,29], and the other half was classified as ‘high risk’ [25] and ‘some concerns’ [21,22]. The remaining one study, which we were able to use only in the systematic review, showed a significant improvement in favor of the control group [3].

**Jebsen Taylor hand function:** The Jebsen Taylor hand function test was examined in four studies [21,23,28,39] (Table 1). Half of them found no differences between the study groups [28,39], and the other two studies showed insignificant improvements [21,23]. The risk of bias was rated ‘low risk’ for three [23,28,39] and ‘some concerns’ for one article [21].

### 3.4. Quantitative Analysis

This meta-analysis included 20 studies and evaluated the upper extremity function with eight outcomes.

**Figure 2:**
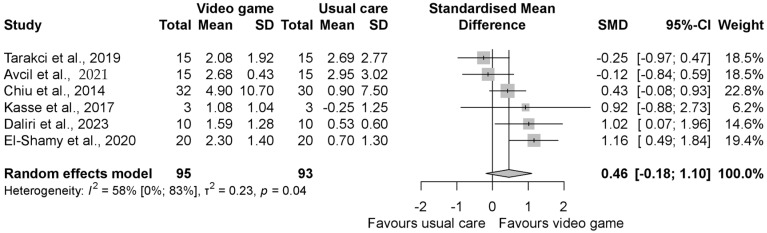
Forest plot of standardized mean differences in grip strength, comparing the intervention video-game-based therapy to other rehabilitation forms (usual therapy, neurodevelopmental treatment, resistance training, and Occupational Therapy). Included articles are: Tarakci et al., 2019 [39], Avcil et al., 2021 [32], Chiu et al., 2014 [23], Kasse et al., 2017. [25], Daliri et al., 2023 [34], El-Shamy et al., 2020 [24].

**Figure 3:**
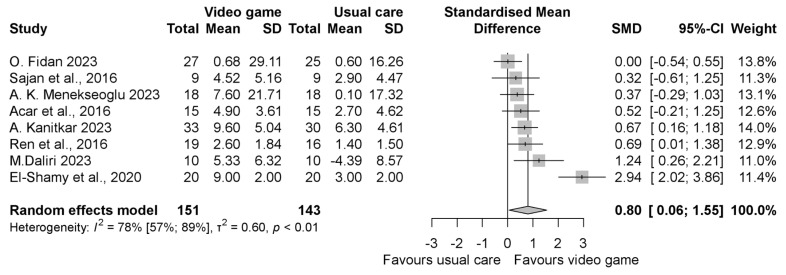
Forest plot of standardized mean differences in grasp function, comparing the intervention video-game-based therapy to other rehabilitation forms (conventional and neurodevelopmental therapy, Occupational Therapy, CIMT, HABIT, and exercise therapy). Included articles are: O. Fidan et al., 2023 [37], Sajan et al., 2016 [17], A.K.Menekseoglu et al., 2023 [26], Acar et al., 2016 [21], A.Katinkar et al., 2023 [38], Ren et al., 2016 [31], M. Daliri et al., 2023 [34], El-Shamy et al., 2020 [24].

**Figure 4:**
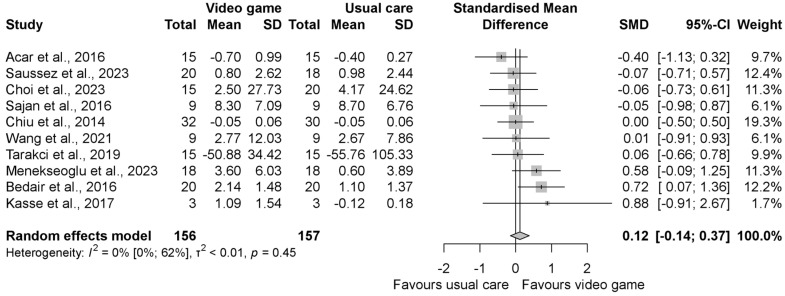
Forest plot of standardized mean differences in manual dexterity physical tests, comparing the intervention video-game-based therapy to other rehabilitation forms (conventional, neurodevelopmental, resistance training and constraint-induced therapy, occupational, HABIT, and exercise therapy). Included articles are: Acar et al., 2016 [21], Saussez et al., 2023 [28], Choi et al., 2023 [33], Sajan et al., 2016 [17], Chiu et al., 2014 [23], Wang et al., 2021 [29], Tarakci et al., 2019 [39], A.K.Menekseoglu et al., 2023 [26], Bedair et al., 2016 [22], Kasse et al., 2017. [25].

**Figure 5:**
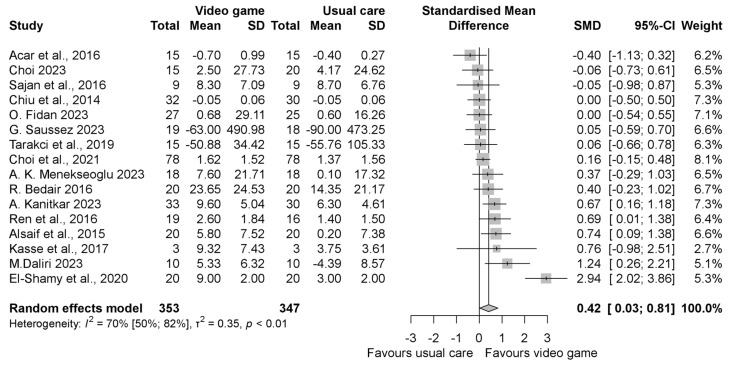
Forest plot of standardized mean differences in upper limb fine motor function tests, comparing the intervention video-game-based therapy to other rehabilitation forms (conventional, neurodevelopmental, resistance training and Occupational Therapy, HABIT, exercise therapy or absence of treatment). Included articles are: Acar et al., 2016 [21], Choi et al., 2023 [33], Sajan et al., 2016 [17], Chiu et al., 2014 [23], O. Fidan et al., 2023 [37], G. Saussez et al., 2023 [28], Tarakci et al., 2019 [39], Choi et al., 2021 [36], A.K.Menekseoglu et al., 2023 [26], R. Bedair et al., 2016 [22], A.Katinkar et al., 2023 [38], Ren et al., 2016 [31], Alsaif et al., 2015 [30], Kasse et al., 2017. [25], M. Daliri et al., 2023 [34], El-Shamy et al., 2020 [24].

**Figure 6:**
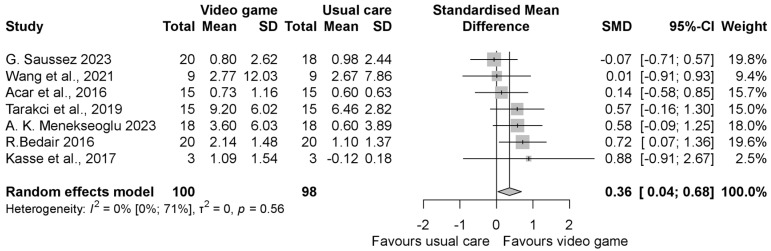
Forest plot of standardized mean differences in hand function questionnaires, comparing the intervention video-game-based therapy to other rehabilitation forms (conventional, neurodevelopmental, resistance training, constraint- induced therapy, HABIT, and exercise therapy). Included articles are: G. Saussez et al., 2023 [28], Wang et al., 2021 [29], Acar et al., 2016 [21], Tarakci et al., 2019 [39], A.K.Menekseoglu et al., 2023 [26], R. Bedair et al., 2016 [22], Kasse et al., 2017. [25].

The pooled standardized mean difference (SMD) regarding the grip strength was insignificantly better in the intervention group compared to the control group with an SMD of 0.46 (95% CI −0.18, 1.10), as shown in Figure 2. When evaluating the result of grasp function, similar to grip strength, it was found to be in favor of the VGBT group, but the result was statistically significant with the SMD 0.80 (95% CI 0.06, 1.55) (Figure 3). In the manual dexterity physical test, the pooled SMD was 0.12 (95% CI −0.14, 0.37), indicating insignificant change (Figure 4). When evaluating the upper limb fine motor function and hand function questionnaires, an improvement with statistically significant differences was seen in both cases; the motor function SMD was 0.42 (95% CI 0.03, 0.81) (see in Figure 5), and the hand function questionnaires favored VGBT with an SMD of 0.36 (95% CI 0.04, 0.68) (Figure 6). Despite these results, the effect of VGBT on the WeeFim (self-care) score was only insignificantly greater than the control group with a mean difference (MD) of 4.22 (95% CI −2.12, 10.55) (Figure S1). As we assessed the Abilhand-Kids and Jebsen Taylor hand function separately, we found a non-significantly greater improvement in the study groups. The MD was 0.60 (95% CI −0.20, 1.39) (Figure S2) in case of Abilhand-Kids, and in Jebsen Taylor hand function, the SMD was 0.07 (95% CI −0.16, 0.31) (Figure S3).

## 4. Discussion

Data reported in the eligible RCTs allowed us to analyze eight different outcomes: grip strength, grasp function, manual dexterity, the Jebsen Taylor hand function, self-care, hand function questionnaire, ABILHAND-Kids results and upper limb fine motor function.

We observed a better improvement in the intervention groups for all outcomes; in three of them (upper limb fine motor function, hand function questionnaire, and grasp function), the difference was statistically significant, and in the remaining five cases (grip strength, WeeFim self-care, AbilHand Kids, the Jebsen Taylor hand function, and manual dexterity), no significant improvement could be detected. Interpretation of standardized mean differences (SMDs) is always challenging; however, the standard interpretation is that a change in grasp function can be considered a large effect. For grip strength, upper limb fine motor function, Weefim self-care, hand function questionnaires and AbilHand Kids, the effect was moderate, whereas in manual dexterity and the Jebsen Taylor hand function, the effect was small.

Handgrip strength is essential for performing various functional activities in everyday life, and it serves as an objective measure of hand function [40]. Muscle weakness in grip strength can be the result of a decrease in the strength of agonists or an imbalance in muscle tone, as seen in cases of spasticity [41]. Some studies find that grip strength is not directly related to the performance of daily activities, whereas others suggest that the non-dominant hand grip strength plays an important role in stabilizing objects [42]. The assessment of grip strength parameters is a commonly used method to determine hand function impairment [41] that does not have a specific cut-off point. Children are affected by cerebral palsy in different ways and will have different grip strengths at different ages. In the research studies, grip strength was measured with a hand dynamometer. Yildirim et al. [43], for example, reported a significant improvement in grip strength after VGBT compared to Structured-Neurodevelopmental-Therapy-based hand rehabilitation in patients with CP. Similarly, a meta-analysis published in 2022 that analyzed three RCTs on the use of the Nintendo Wii alone or in combination revealed a significant increase in grip strength compared to traditional rehabilitation methods [44]. In contrast, our current work found no significant difference between the groups. Taking the results into consideration that in all articles, both VGBT or any other conventional treatment improved grip strength (with the exception of one study [25]), we believe that the moderate, non-significant difference in favor of VGBT alone or in combination with other forms of rehabilitation means that this form of treatment is not superior to other therapeutic forms of rehabilitation. When evaluating the results, it is worth considering the heterogeneity of the applied therapy in the control group; due to the small number of articles, we could not perform a subgroup analysis.

Although there is no consensus on the relationship between grip strength and functional activities, children with CP usually have muscle weakness, spasticity and coordination problems, which can lead to a significant loss of reaching and grasping function [41,45]. A meta-analysis on the effectiveness of Nintendo Wii found that the continuous grip of the joystick can increase grip strength and improve grasping ability. A limitation of this study is that Nintendo Wii was used as a supplementary therapy, as Nintendo Wii + conventional therapy as an intervention group was compared to conventional therapy alone as a control group [44]. In order to evaluate grasp function statistically, we combined the Peabody grasp and the QUEST grasp subdomains. In line with previous articles, we found significant improvements after using the video game alone or in combination with other therapy compared to control groups, suggesting that the effectiveness of VGBT alone or as an adjunctive therapy may be potentially greater in improving hand grasp function than traditional forms of therapy alone. Although maximal voluntary contraction may not be reflected in muscle coordination, it may be equally important in the performance of manual tasks during daily activities [41]. Considering the number of examined articles and the heterogeneity of the control group, we could not perform further statistical subgroup analysis.

In order to better understand the mechanism of the therapy and how the therapy can have an effect on daily activities through coordination, we investigated manual dexterity tests. A deficiency in manual dexterity of the affected limb is often seen in children with cerebral palsy. In a non-RCT study, a significant improvement in the Jebsen Taylor hand function test, measuring manual dexterity, was observed after VGBT [43]. Our findings on VGBT are consistent with a previous meta-analysis that investigated the use of Nintendo-based therapy alone or as an adjunct therapy to improve manual dexterity, as we observed no significant difference between the two groups examined, even when we compared only the Jebsen Taylor hand function tests [44]. Despite the fact that most of the studies assessing the effect of video-game-based therapy on manual dexterity used video games controlled by a leap motion sensor, which mainly uses larger proximal joint movements rather than fine motor movements, our findings demonstrate that there is no significant difference between this therapy and standard therapies in improving manual dexterity. In order to better understand the effect of VGBT, we could not perform further statistical calculations for the intervention or control group.

In order to assess the effect of this innovative therapy on upper limb motor function as thoroughly as possible, one physical test from each paper was selected, results were pooled, and the SMD between groups was calculated. The authors discussed and selected one test from each paper that reflected the upper limb motor function. For multiple tests, we used the one that showed the most cases. Using this method, in contrast to our previous findings, we observed a significant improvement in favor of VGBT used alone or in combination with other forms of rehabilitation compared to other rehabilitation forms, suggesting that VGBT as a supplementary therapy can have benefits over conventional therapeutic forms alone.

To determine whether the improvement shown by the functional tests corresponds to a similar change indicated by the upper limb function questionnaires, we decided to evaluate the upper limb questionnaires separately from the functional tests. The questionnaires included Abilhand kids and the Duruoz hand index, which focus mainly on daily activity [3]. A crossover trial that we were only able to use in the systematic review of CP children showed that conventional therapy proved to be more effective in improving manual abilities in daily activities, evaluated by Abilhand [3]. Conversely, another RCT study of patients with juvenile idiopathic arthritis using the Duruoz hand index found that video-game-based therapy was better than the control group [46]. Our analysis of questionnaires assessing upper extremity function showed that VGBT alone or in combination with other rehabilitation techniques resulted in significantly greater improvement than other rehabilitation techniques. When we analyzed the Abilhand-Kids questionnaires individually, intervention groups showed insignificantly better improvement than the control groups.

We investigated the self-care function of Weefim to see if there was any change in everyday activities as a result of the other physical and questionnaire tests [6]. In a crossover study, a greater improvement in upper limb motor skills was observed, but this represented no significant improvement in everyday activities compared to the control group [3]. Similarly to the meta-analysis that examined only the therapeutic effect of Nintendo Wii on daily activities and self-care function, our study also found that VGBT alone or as an adjunctive therapy was equally effective compared to the control groups [44].

In an article by El-Shamy, significant improvements were observed in three out of eight outcomes (grip, grasps, and fine motor function of the upper limb), as opposed to the results of our meta-analysis. One possible interpretation of this result is that, in the study by El-Shamy et al., the improvement in the intervention group may have been due to an increased amount of time (additional 24 h) in the study group (Nintendo Wii+ usual care) compared to the control group (usual care).

It is important to highlight that in our endeavor to comprehensively summarize and analyze the data available in the literature, we had to accept some clinical and methodological heterogeneity among the included papers. The included studies differed in the type of VGBT applied, the games played, and in the interventions applied in the comparator group. However, we decided to proceed with a meta-analysis for several reasons. For one, we hoped to discover potential sources of heterogeneity by visual inspection of Forest plots, which could be the source of further sensitivity testing and subgrouping if a hypothesis could be established (such as seeing a superiority of VGBT over some forms of comparator, while not over others). We were unable to establish any such hypothesis, but reported details of the studies alongside our results to provide an opportunity to the reader to attempt the same investigation. Secondly, children with cerebral palsy (CP) in different age groups and with varying degrees of impairment often receive different forms of “usual care” influenced by their life circumstances, healthcare systems, and available resources. While greater homogeneity in the control group would enhance the scientific rigor of the study, we believe that the current diversity better reflects the variety of therapies employed in daily practice. We think that the key limitation, however, is the heterogeneity within the intervention group. Each game can produce distinct effects, as they involve repetitive execution of various movements, even when using the same device. This variability is further amplified when different tools are used, which underscores the need for more standardized study designs. For example, in Katinkar’s 2023 article, a joystick resembling a small tennis ball was described as less effective in promoting finer movements and hand fine motor skills than other video-game-based therapy devices. On the Nintendo Wii, hand movements are generally larger, but pressing buttons still requires finger dexterity. The REAtouch^®^ device, on the other hand, eliminates the need to press buttons. Instead, it requires users to grasp a variety of objects, which has less of an impact on fine motor skills but may impact the development of grip strength. In systems like the leap motion controller, as Daliri et al. noted, hand gestures play a prominent role in interaction. Continuous use of a controller during games such as VR or Wii can improve grip strength to a greater extent than fine motor skills. Despite these challenges, our aim was to address the limited and diverse range of existing studies by aggregating their effects, acknowledging these limitations, and attempting to provide insights into these therapies through statistical analysis.

The need for new innovative forms of rehabilitation techniques that has emerged with the development of new technology is understandable, and our findings suggest that video-game-based therapy may be a beneficial adjunctive therapy in the daily upper extremity rehabilitation of children with cerebral palsy.

### 4.1. Implications for Practice and Policy Makers

The timely application of scientific results at the bedside is of paramount importance [47,48]. Due to the potential benefits of video-game-based therapy and the absence of reported harmful effects, it may be worth considering the use of this treatment as an additional tool to other traditional therapies. The establishment of guidelines or at least institutional protocols for the integration of VGBT into upper extremity rehabilitation in children with CP may lead to better compliance and, therefore, improved therapeutic outcomes. Moreover, a new therapy of real interest to children with cerebral palsy could be introduced.

### 4.2. Implications for Research

The results of our analysis suggest that further investigations should be conducted in which only VGBT is included to investigate its independent effect. More homogeneous outcomes would be needed to gain more insight into the detailed mechanism of action of this therapy. In order to understand the mechanism of action in the rehabilitation of video-game-based therapy, it is necessary to distinguish between leap motion video-game-based therapy and controller-based video game. Additionally, considering the limitations of our study, we propose that these investigations should initiate long-term studies and should also take into account the type of video game. Investigators should take care to mitigate biasing factors such as increased intervention time in the intervention group or combination therapy.

### 4.3. Strengths and Limitations

The strength of our work is that it is the most recent and most comprehensive meta-analysis available in the literature. We included only RCTs, making it the highest level of evidence to date.

We were also able to pool data from different tests.

Given the large number of patients and the diverse demographics, the findings can be broadly applied to children diagnosed with cerebral palsy (CP).

Some limitations that could potentially influence the results of our meta-analysis should be highlighted: the majority of the articles had short investigation periods (3–12 weeks). Studies of longer duration would be necessary to develop a more accurate picture of these new methods. Another limiting factor is the heterogeneity of intervention types (VGBT+ other forms of rehabilitation) and the heterogeneity of the therapy applied in the control groups (for example, occupational, traditional, therapy, and constraint-induced therapy). In an article [17], the age range was 5–20, but the mean age with SD was below 18 years in the control and in the investigation group; however, considering the low number of cases, we decided to include it.

Some articles used different treatment durations between intervention and control groups.

Different measurement methods were used, making statistical analysis problematic, sometimes even impossible.

The results for the grasp function and upper limb fine motor function are potentially distorted by the article by El-Shamy et al., where the intervention group received 40% more session time compared to the control group; therefore, these significant results should be treated with a sufficiently critical attitude.

## 5. Conclusions

Our results suggest that VGBT rehabilitation may be an effective tool for rehabilitation of disabled upper limb function in cerebral palsy. In terms of upper limb motor function tests and questionnaire and grasp function, this new form of treatment, when complemented with other therapies, is superior to conventional forms of rehabilitation alone.

## Figures and Tables

**Table 1:** Results from each article.

Outcomes	Included Articles	Unite of Measurement	Intervention	Control	Favours
**Grip Strength Grade: High**	Chiu et al. 2014 [23]	Newton (hand dynamometer)	Nitendo Wii	Usual Therapy	Intervention
	M.Daliri et al. 2023 [34]	Kg (hand dynamometer)	LMC	Occupational therapy	Intervention *
	El-Shamy et al. 2020 [24]	Kg (hand dynamometer)	Nintendo Wii + Usual care	Usual care	Intervention *
	Kassee et al. 2017 [25]	pounds per square inch (psi)(hand dynamometer)	Nintendo Wii	Resistance training	Intervention
	E.Avcil et al. 2020 [32]	Kg (hand dynamometer)	Nitendo Wii + LMC	Neurodevelopmental training	equal
	Tarakci et al. 2019 [39]	LB (hand dynamometer)	LMC	Conventional treatment	Control
**Grasp function Grade: Moderate**	O.Fidan et al. 2023 [37]	QUEST Grasps domain score	VR int.	Neurodevelopmental training	equal
	Sajan et al. 2016 [17]	QUEST Grasps domain score	Ninetendo Wii + conventional th.	conventional th.	Intervention
	A.K.Menekseoglu et al. 2023 [26]	QUEST Grasps domain	VR int.+ Exercise th.	Exercise th.	Intervention
	Acar et al. 2016 [21]	QUEST Grasps domain	Nintendo Wii + NDT	Neurodevelopmental training	Intervention
	A.Katinkar et al. 2023 [38]	PMDS-2 grasp subdomain	Computer Games	CIMT and HABIT	Intervention *
	Ren et al. 2016 [31]	PMDS-2 grasp subdomain	VR int.	conventional th.	Intervention *
	M.Daliri et al. 2023 [34]	QUEST Grasps domain	LMC	Occupational therapy	Intervention *
	El-Shamy et al. 2020 [24]	PMDS-2 grasp subdomain	Nintendo Wii + Usual care	Usual care	Intervention *
**Manual Dextertiy Grade: Moderate**	Acar et al. 2016 [21]	Jebsen Taylor Hand Function Test	Nintendo Wii+NDT	Neurodevelopmental training	Control
	Saussez et al. 2023 [28]	ABILHAND-Kids	REAtouch+HABIT-ILE	HABIT-ILE	equal
	Choi et al. 2023 [33]	ABILHAND-Kids	VR int.	Occupational therapy	equal
	Sajan et al. 2016 [17]	Box and Block Test	Ninetendo Wii + conventional th.	conventional th.	equal
	Chiu et al. 2014 [23]	Jebsen Taylor Hand Function Test	Nitendo Wii	Usual Therapy	equal
	Wang et al. 2021 [29]	ABILHAND-Kids	Nintendo Wii +Constraint-induced th.	Constraint-induced th.	equal
	Tarakci et al. 2019 [39]	Jebsen Taylor Hand Function Test	LMC	Conventional treatment	equal
	A.K.Menekseoglu et al. 2023 [26]	ABILHAND-Kids	VR int.+ Exercise th.	Exercise th.	Intervention
	Bedair et al. 2016 [22]	ABILHAND-Kids	VR int.+ physical th.	physical therapy	Intervention *
	Kassee et al. 2017 [25]	ABILHAND-Kids	Nintendo Wii	Resistance training	Intervention
**Jebsen Taylor Hand Function Grade: High**	Saussez et al. 2023 [28]	Jebsen Taylor Hand Function Test	REAtouch+HABIT-ILE	HABIT-ILE	equal
	Tarakci et al. 2019 [39]	Jebsen Taylor Hand Function Test	LMC	Conventional treatment	equal
	Chiu et al. 2014 [23]	Jebsen Taylor Hand Function Test	Nitendo Wii	Usual Therapy	Intervention
	Acar et al. 2016 [21]	Jebsen Taylor Hand Function Test	Nintendo Wii+NDT	Neurodevelopmental training	Intervention
**Upper Limb Fine Motor Function Grade: Moderate**	Acar et al. 2016 [21]	jebsen taylor hand function	Nintendo Wii+NDT	Neurodevelopmental training	Control
	Choi et al. 2023 [33]	Melbounre assesment II.	VR int.	Occupational therapy	equal
	Sajan et al. 2016 [17]	Box and Block Test	Ninetendo Wii + conventional th.	conventional th.	equal
	Chiu et al. 2014 [23]	jebsen taylor hand function	Nitendo Wii	Usual Therapy	equal
	O.Fidan et al. 2023 [37]	QUEST Grasps domain	VR int.	Neurodevelopmental training	equal
	Saussez et al. 2023 [28]	Jebsen Taylor Hand Function Test	REAtouch+HABIT-ILE	HABIT-ILE	equal
	Tarakci et al. 2019 [39]	jebsen taylor hand function	LMC	Conventional treatment	equal
	Choi et al. 2021 [36]	ULPRS	Virtual int. + Occupational therapy	Occupational therapy	equal
	A.K.Menekseoglu et al. 2023 [26]	QUEST Grasps domain	VR int.+ Exercise th.	Exercise th.	Intervention
	Bedair et al. 2016 [22]	PMDS-2	VR int.+ physical th.	physical therapy	Intervention
	A.Katinkar et al. 2023 [38]	PMDS-2	Computer Games	CIMT and HABIT	Intervention *
	Ren et al. 2016 [31]	PMDS-2	VR int.	conventional th.	Intervention *
	Alsaif et al. 2015 [30]	Movement Assessment Battery for Children-2 (mABC-2)	Nintendo Wii	No training	Intervention *
	Kassee et al. 2017 [25]	Melbounre assesment II.	Nintendo Wii	Resistance training	Intervention
	M.Daliri et al. 2023 [34]	QUEST Grasp domain	LMC	Occupational therapy	Intervention *
	L.Zoccolillo 2016 [3]	QUEST score	LMC	conventional th.	
	El-Shamy et al. 2020 [24]	PMDS-2	Nintendo Wii + Usual care	Usual care	Intervention *
**Hand Function Questionnaires Grade: Moderate**	Saussez et al. 2023 [28]	ABILHAND-Kids	REAtouch+HABIT-ILE	HABIT-ILE	equal
	Wang et al. 2021 [29]	ABILHAND-Kids	Nintendo Wii +Constraint-induced th.	Constraint-induced th.	equal
	Acar et al. 2016 [21]	ABILHAND-Kids	Nintendo Wii+NDT	Neurodevelopmental training	equal
	Tarakci et al. 2019 [39]	Duruoz Hand Index	LMC	Conventional th.	Intervention
	A.K.Menekseoglu et al. 2023 [26]	ABILHAND-Kids	VR int.+ Exercise th.	Exercise th.	Intervention
	Bedair et al. 2016 [22]	ABILHAND-Kids	VR int.+ physical th.	physical therapy	Intervention *
	Kassee et al. 2017 [25]	ABILHAND-Kids	Nintendo Wii	Resistance training	Intervention
**ABILHAND-Kids Grade: High**	L.Zoccolillo 2016 [3]	ABILHAND-Kids	LMC	conventional th.	Control *
	Saussez et al. 2023 [28]	ABILHAND-Kids	REAtouch+HABIT-ILE	HABIT-ILE	equal
	Wang et al. 2021 [29]	ABILHAND-Kids	Nintendo Wii +Constraint-induced th.	Constraint-induced th.	equal
	Acar et al. 2016 [21]	ABILHAND-Kids	Nintendo Wii+NDT	Neurodevelopmental training	equal
	A.K.Menekseoglu et al. 2023 [26]	ABILHAND-Kids	VR int.+ Exercise th.	Exercise th.	Intervention
	Bedair et al. 2016 [22]	ABILHAND-Kids	VR int.+ physical th.	physical therapy	Intervention *
	Kassee et al. 2017 [25]	ABILHAND-Kids	Nintendo Wii	Resistance training	Intervention
**Self Care (WeeFim). Grade: Moderate**	Tarakci et al. 2016 [35]	WeeFim selfcare domain	Nintendo Wii	conventional th.	Intervention
	Sahin et al. 2019 [27]	WeeFim selfcare domain	VR int.+ TOT	TOT	Intervention
	Acar et al. 2016 [21]	WeeFim selfcare domain	Nintendo Wii+NDT	Neurodevelopmental training	Intervention *

Kg—kilogram; LB—pound; QUEST—Quest quality of upper extremity skills test; PMDS-2—Peabody Developmental Motor Scales-2; ULPRS—Upper Limb Physician’s Rating Scale; WeeFim—Functional Independence Measure for Children; LMC—leap motion controller; VR int.—virtual reality intervention; Exercise th.—exercise therapy; NDT—neurodevelopmental treatment; HABIT-ILE—Hand Arm Bimanual Intensive Training Including Lower Extremity; Conventional th.—conventional therapy; Constraint-induced th.—constraint-induced therapy; Physical th.—physical therapy; CIMT—Constraint-Induced Movement Therapy, * significant difference.

## Supplementary Materials

The following supporting information can be downloaded at: https://www.mdpi.com/article/10.3390/jcm13247524/s1, Table S1: Baseline characteristics of studies included; Table S2: Results of the risk of bias assessment using the Cochrane Risk of Bias tool, version 2; Figure S1: Forest plot of Mean Differences in Self Care (WeeFim), comparing the intervention video game-based therapy to other rehabilitation forms (conventional therapy, neurodevelopmental therapy, and occupational therapy); Figure S2: Forest plot of Mean Differences in ABILHAND-Kids, comparing the intervention video game-based therapy to other rehabilitation forms (conventional, neurodevelopmental, resistance training, constrain induced therapy, and exercise therapy). Figure S3: Forest plot of Standardized Mean Differences in the Jebsen Taylor Hand Function, comparing the intervention video game-based therapy to other rehabilitation forms (conventional, neurodevelopmental, and HABIT.

## Abbreviations and Acronyms

CI—confidence interval; CIMT—Constraint-Induced Movement Therapy; CP—cerebral palsy; CT—conventional treatment; ET—exercise therapy; GRADE—Grading of Recommendations, Assessment, Development, and Evaluations; HABIT—Hand Arm Bimanual Intensive Therapy; LMC—leap motion controlled; NDT—neurodevelopmental training; OT—Occupational Therapy; QUEST—Quality of upper extremity skills test; RCT—randomized controlled trial; ROB2—risk-of-bias tool for randomized trials; RT—resistance training; SD—standard deviation; SMD—standardized mean difference; VGBT—video-game-based therapy.
