# Supplementary material for: Effectiveness of Video-Game-Based Therapy to Improve Hand Function in Children with Cerebral Palsy: A Systematic Review and Meta-Analysis
Source: J Clin Med. 2024 Dec 11;13(24):7524. doi: 10.3390/jcm13247524 (PMC11728108; doi:10.3390/jcm13247524)
Supplement: Supplementary file 1 [file jcm-13-07524-s001.zip › jcm-3325187-supplementary.pdf]

Journal of
Clinical Medicine

MDPI

Supplementary Table S1. Baseline characteristics of studies included.

F. Author/Pub. Year	Country	Patient N.	Mean Age (Years)	Female %	Duration/ Intensity	Study Groups	comparator	Results Found	Outcome	Other information
**S.Sahin/2019**	Turkey	60	I:10.5 +-3.62 C: 10.06+- 3.24	38,3%	45min.x2d.x8w	VR + (TOT)	TOT	VR improves motor skills and independence.	WeeFim Selfcare	Game:Air challenge,Boxing trainer,Wall breaker,Jet run,Super kick.
**G.Acar/2016**	Turkey	30	I:9,53+-3,04 C:9,73+-2,86	46,6%	45min.x2dx6 w.	N.Wii + NDT	NDT	NDT improves hand function in hemi CP.	QUEST Grasp,JTHFT,ABILHAND-Kids,WeeFim	Games:tennis, baseball, and boxing.
**A.Alsaif/2015**	Saudi Arabia	40	6-10	Ni.	20min.x7d.x12w.	N.Wii	No training	The Nintendo Wii significantly improved motor tests.	m-ABC-2	[illegible] arm. movements, balancing, and jumping
**S. Atasavun/2016**	Turkey	24	I:9,13+-2,57 C:10,11+-2,62	58,3%	30min.x2d.x12w.	N.Wii + Physio Th.	Physio Th.	Nintendo Wii improved occupational performance, daily activities, and balance.	PEDI	Games: basketball, tennis, boxing.
**E. Avcil/2020**	Turkey	30	I:10,93+-4,09 C:11,07+-3,24	43,4%	24 session/8 w.	N.Wii + LMC	NDT	LMC games slightly improved manual dexterity in CP patients.	Grip Strength	Games: Fizyosoft®CatchAPet, Leapball.
**H. C. Chiu/2014**	Taiwan	62	I:9,4+-1,9 C:9,5+-1,9	54,8%	40min.x3d.x6 w	N.Wii + Usual Th.	Usual Th.	Wii™ training showed no improvement in coordination, strength, or hand function.	JTHFT,Grip strength	Game: Wii Sports Resort.
**S. M. El-Shamy/2018**	Egypt	40	I:9,5+-1,2C:9,8+-1,4	35%	40min.x3d.x12 w.	N.Wii + Usual Th.	Usual Th.	Wii training with usual care reduces spasticity and improves grip strength and hand function.	PMDS-2,Grip strength, PMDS-2 grasp	Games:tennis;boxing;bowling; basketball.
**J. Y. Choi/2021**	Korea	78	5,66+-2,83	51,28%	60min.x5d.x4 w.	VR+OT	OT	Virtual reality rehabilitation is effective for children with brain injury.	ULPRS	Game: RAPAEL Smart Kids.
**C. Kassee/2017**	Canada	6	9,33	0%	40min.x5d.x6w.	N.Wii	Resistance tr.	Wii training may be an effective home rehabilitation strategy.	Grip strength,ABILHAND-Kids, Melbourne Assesment II.	Games: Wii Nunchuck,Wii Sports Resort.
**T. N. Wang/2021**	Taiwan	18	I:8,55+-2,09C:8,56+-2,15	61%	135min.x2d.x8w.	N.Wii + CIT	Const.-ind. Th.	CIT-Wii shows no significant difference in effects compared to conventional CIT.	ABILHAND-Kids	Games: e.g.,Sports Resort, Wii Sports, Mario Sports Mix, Cooking Mama: Cook Off, Let's Tap, and Happy Dance Collection.
**J. E. Sajan/2016**	India	18	I:12,4+-3,78C:12,4+-4,93	45%	45min.x6dx3w.	N.Wii	CT	The intervention group showed significant improvement in upper limb function.	Quest,BBT	Games: boxing, tennis.
**D. Tarakci/2016**	Turkey	30	I:10,46+-2,69C:10,53+-2,79	36,6%	50min.x2d.x12w.	N.Wii + NDT	CT	Video games, combined with NDT, better improve static and performance-related balance.	WeeFim	Games: walking on rope,skiing,Tilt Table-Balance Board,Heading
**D. Tarakci/2019**	Turkey	30	I:10,93+-4,09C:11,06+-33,23	43,3%	60min.x3d.x8w.	LMC	CT	LMCBT should be an effective alternative treatment for children with physical disabilities.	Grip strength, JTHFT,Duruoz Hand index	Fizyosoft Games: CatchAPet,Leapball.
**K. Ren/2016**	China	35	I:4,75+-0,83C:4,5+-1,16	42,8%	40min.x5d.x12w.	VR + (OT)	CT+OT	VR training effectively improves fine motor function in the upper limbs.	PMDS-2,Grip strength, PMDS-2 grasp	-
**O.Fidan/2023**	Turkey	52	I:9,2+-2,08 CT:9,4+-2,25	40,38%	45min.x2dx8 w.	VR	NDT	Kinect-based VR training helps improve balance, motor function, and upper extremity skills.	QUEST	Games:fruits Ninja,tennis game, soccer,bowling.
**A. K. Menekseoglu/2023**	Turkey	36	I:8,2+-1,8C:8,3+-1,4	47,2%	60min.x2d.x6w.	VR + Exercise th.	Exercise th.	Virtual reality improved upper limb function, quality of life, and active joint range of motion in children with hemiplegic cerebral palsy.	QUEST, ABILHAND-Kids	Games: butterfly,bee,eagle game.
**A. Kanitkar/2023**	India	63	I:7,3+-2,1C:7,8+-1,9	-	45min.x3d.x16w.	PC games-based e.	CIMT + HABIT	This study shows a positive effect of GRP.	PMDS-2	Games:arcade-style.
**R.Bedair/2016**	Egypt	40	I:7,05+-0,99C:7,25+-0,96	42,5%	60min.x3d.x16w.	VR + Physical Th.	Physio Th.	Significant improvement in upper limb function in the study group post-treatment is linked to the active participation of children in a simulated environment.	ABILHAND-Kids, PMDS-2	Games: tennis, bowling, golf, space pop, bubbles,boat driving.
**G. Saussez/2023**	Belgium	38	I:9+-3,1C:9,1+-2,9	50%	90h/2w	REAtouch +HABIT-ILE	HABIT-ILE	The use of the REAtouch® device during HABIT-ILE demonstrated similar efficacy to the conventional evidence-based HABIT-ILE intervention.	ABILHAND-Kids, JTHFT	Regular Games: (e.g., board games, card, building activities, etc.)
**M. Daliri/2023**	Iran	20	I:6,4+-1,07C:6,1+-1,1	25%	60min.x2d.x16w.	LMC	OT	Adding LMC to the rehabilitation program for CP patients may improve upper extremity motor function.	QUEST, Grip Strength	Games:cube grasping, flower petal,removal,Kyoto.
**J. Y. Choi/2023**	Italy	35	I:8,1+-3,2C:7,3+-2,6	48,6%	30min.x5.dx6w.	VR + (OT)	OT	Home-based VR training had a limited impact on improving upper limb function.	ABILHAND-Kids, Melbourne Assesment II.	Game: RAPAEL Smart Kids.
**L.Zoccolio/2016**	Italy	18	6,89+-1,91	-	90min.x2d.x8 weeks	Xbox kinect+ Usual Th.	CT	VGT was effective in improving upper limb motor function	QUEST, ABILHAND-Kids	Game:Xbox kinect adventure package.

[1] I.[2] C.[3]min.[4]d.[5]w.[6]VR[7]TOT[8]N.Wii[9]NDT[10]Physio th.[11]LMC[12]Usual Th.[13]OT[14]CIT[15]exercise th.[16]PC games-based e.[17] Physical Th.[18]HABIT-ILE[19]Resistance tr.[20]Const-ind Th.[21]CT[22]CIMT[23]HABIT[24]WeeFim[25]QUEST[26]JTHFT[27]m-ABC-2[28]PEDI[29]PMDS-2[30]ULPRS[31]BBT

[1] Intervention

[2] Control group

[3] Minute

[4] Day

[5] week

[6] Virtual reality

[7] Traditional occupational therapy

[8] Ninetendo Wii

[9] Neurodevelopmental treatment

[10] Physio therapy

[11] Leap motion controller

[12] Usual therapy

[13] Occupational therapy

[14] constraint-induced therapy

[15] exercise therapy

[16] Computer game-based therapy

[17] Physical therapy

[18] Hand Arm Bimanual Intensive Training Including Lower Extremity

[19] Resistance training

[20] Constraint induced therapy

[21] Conventional therapy

[22] Constraint induced movement therapy

[23] Hand Arm Bimanual Intensive Training

[24] Functional Independence Measure for Children

[25] Quest quality of upper extremity skills test

[26] Jebsen Taylor Hand Function Test

[27] Movement Assessment Battery for Children-2

[28] Pediatric Evaluation of Disability Inventory

[29] Peabody Developmental Motor Scales-2

[30] Upper Limb Physician's Rating Scale

[31] Box and Block Test

Supplementary Table S2. Results of the risk of bias assessment using the Cochrane Risk of Bias tool, version 2.

Studies with intention-to-treat	Unique ID	Study ID		Comparator	Outcome	Weight	Randomization process	Deviations from intended interventions	Missing outcome data	Measurement of the outcome	Selection of the reported result	Overall
	A1	E.Avcil 2020	Videogame-beased	Neurodevelopmen	Grip Strength	1	+	+	+	+	+	+
	A2	Chiu 2014	Nintendo Wii	Usual therapy	Grip Strength	1	+	+	+	+	+	+
	A3	El-shamy 2018	Nintendo Wii	Usual care only	Grip strength	1	+	+	+	+	+	+
	A4	Kasse 2017	Wii training	Resistance training	Grip Strength	1	–	+	+	+	+	–
	A5	Taracki 2019	Leap motion contr	conventional treat	Grip strength	1	+	+	+	+	+	+
	B1	Sahin 2019	Virtual reality trair	Traditional occupa	WeeFim Selfcare su	1	+	+	+	+	+	+
	B2	G.Acar 2016	Nintendo Wii + neu	Neurodevelopmen	WeeFIm Sefcare do	1	?	+	+	+	+	!
	B3	Tarakci 2016	Ninetendo Wii + ne	Neurodevelopmen	WeeFim selfcare dc	1	–	+	+	+	+	–
	C1	G.Acar 2016	Nintendo Wii+NDT	Neurodevelopmen	Quest Grasp domai	1	?	+	+	+	+	!
	C2	Sajan 2016	Ninetendo Wii + cc	convenctional ther	Quest Grasp domai	1	+	+	+	+	+	+
	C3	El-Shamy 2020	Nintendo Wii + usu	Usual care	PDmS-2 Grasp dom	1	+	+	+	+	+	+
	C4	Ren 2016	Virtual reality trair	Conventional train	PDMS-2 grasp dom	1	?	+	+	+	?	!
	D1	Tarakci 2019	Leap motion contr	Conventional thera	Duruoz Hand Index	1	+	+	+	+	+	+
	D2	G.Acar 2016	Nintendo Wii+NDT	Neurodevelopmen	AbilHand-Kids	1	?	+	+	+	+	!
	D31	Kasse 2017	Wii traning	Resistance traning	AbilHand-Kids	1	–	–	+	?	+	–
	D4	Wang 2021	Nintendo Wii +Cor	Constraint-inducec	AbilHand-Kids	1	+	+	+	+	+	+
	E1	G.Acar 2016	Nintendo Wii+NDT	Neurodevelopmen	Jebsen Taylor Hand	1	?	+	+	+	+	!
	E21	Chiu 2014	Nintendo Wii	Usual Therapy	Jebsen Taylor Hand	1	+	+	+	+	+	+
	E3	Tarakci 2019	Leap motion contr	Conventionak treat	Jebsen Taylor Hand	1	+	+	+	+	+	+
	E4	Sajan 2016	Nitendot Wii+ Con	Conventional thera	Box and Blocks test	1	+	+	+	+	+	+
	E5	Kasse 2017	Nitendo Wii	Resistance training	Melbourne Assessn	1	–	+	+	?	+	–
	E6	Choi 2021	Virtual reality + CO	Conventional occu	ULPRS	1	+	+	+	+	+	+
	E7	Aalsaif 2015	Nintendo Wii	No training	m-ABC-2	1	?	+	+	?	+	!
	D5	R.Bedair-2016	physical therapy+v	physical therapy	AbilHand kids	1	+	+	+	+	?	!
	C6	O.Fidan-2023	Virtual reality trair	Neurodevelopmen	QUEST grasp subdo	1	+	+	+	+	+	+
	C7	A.Katinkar 2023	Computer Games	CIMT and HABIT	PMDS" grasp subdc	1	+	+	+	+	+	+
	A6	M.Daliri 2023	LMC games	Occupational thera	Grip strength	1	+	+	+	+	+	+
	C8	M. Daliri 2023	LMC games	Occupational thera	QUEST GRASP	1	+	+	+	+	+	+
	D6	G. Saussez 2023	REAtouch+HABIT-II	HABIT-ILE	Abilhand-Kids	1	+	+	+	+	+	+
	D7	Choi 2023	VR rehabilitation (I	Occupational Ther	Abilhand-Kids	1	+	+	+	+	+	+
	C5	A.K.Menekseoglu	VR therapy+ Exerci	Exercise therapy	QUEST Grasp subdc	1	+	+	+	+	+	+
	D8	A.K.Menekseoglu	VR tehrapy+ exerci	Exercise therapy	Abilhand-kids	1	+	+	+	+	+	+
	F1	R.Bedair	Virtual reality+ phy	Physical therapy	PMDS2	1	+	+	+	+	?	!
	F2	Choi 2023	VR rehabilitation	Occupational thera	Melbourne Assessn	1	+	+	+	+	+	+
	F3	G.Saussez 2023	REAtouch+ HABIT-I	HABIT-ILE	Jebsen Taylor Hand	1	+	+	+	+	+	+
	D9	L.Zoccolillo 2016	video-game based t	conventional thera	Abilhand-Kids	1	+	+	+	+	+	+
	C9	L.Zoccolilo 2016	Video-game based	Conventional thera	QUEST score	1	+	+	+	+	+	+

[32]NDT[33]LMC[34]HABIT-ILE[35]VR[36]CIMT[37]WeeFim[38]QUEST[39]PMDS-2[40]ULPRS[41]mABC-2

[32] Neurodevelopmental treatment

[33] Leap motion controller

[34] Hand Arm Bimanual Intensive Training Including Lower Extremity

[35] Virtual Reality

[36] Constraint induced movement therapy

[37] Functional Independence Measure for Children

[38] Quest quality of upper extremity skills test

[39] Peabody Developmental Motor Scales-2

[40] Upper Limb Physician's Rating Scale

[41] Movement Assessment Battery for Children-2

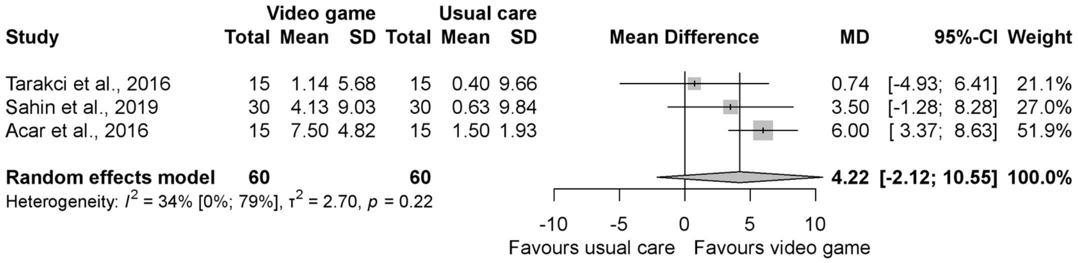

Supplementary Figure S1. Forest plot of Mean Differences in Self Care (WeeFim), comparing the intervention video game-based therapy to other rehabilitation forms (conventional therapy, neurodevelopmental therapy, and occupational therapy).

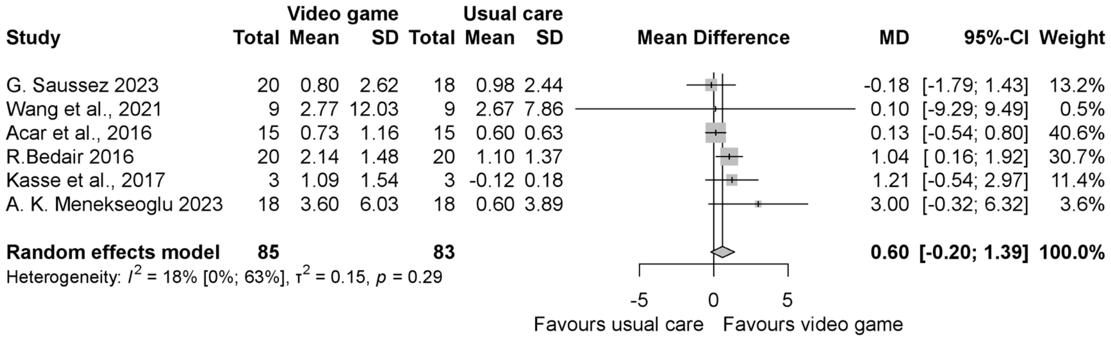

Supplementary Figure S2. Forest plot of Mean Differences in ABILHAND-Kids, comparing the intervention video game-based therapy to other rehabilitation forms (conventional, neurodevelopmental, resistance training, constrain induced therapy, and exercise therapy).

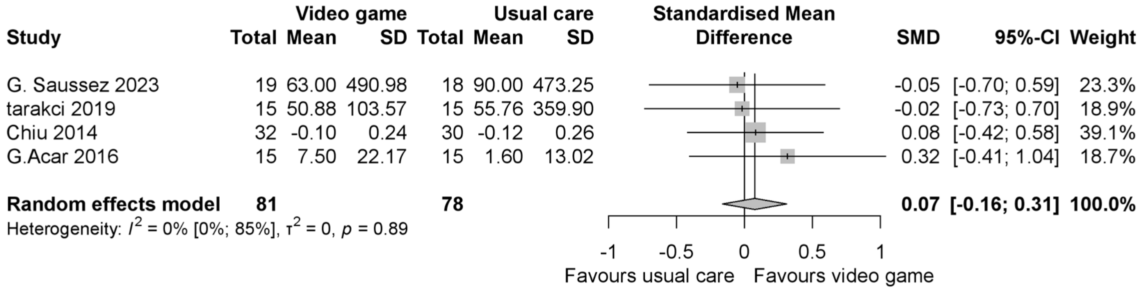

Supplementary Figure S3. Forest plot of Standardized Mean Differences in the Jebsen Taylor Hand Function, comparing the intervention video game-based therapy to other rehabilitation forms (conventional, neurodevelopmental, and HABIT.
